# Prediction of Drug Side Effects with a Refined Negative Sample Selection Strategy

**DOI:** 10.1155/2020/1573543

**Published:** 2020-05-09

**Authors:** Haiyan Liang, Lei Chen, Xian Zhao, Xiaolin Zhang

**Affiliations:** ^1^College of Information Engineering, Shanghai Maritime University, Shanghai 201306, China; ^2^Shanghai Key Laboratory of PMMP, East China Normal University, Shanghai 200241, China

## Abstract

Drugs are an important way to treat various diseases. However, they inevitably produce side effects, bringing great risks to human bodies and pharmaceutical companies. How to predict the side effects of drugs has become one of the essential problems in drug research. Designing efficient computational methods is an alternative way. Some studies paired the drug and side effect as a sample, thereby modeling the problem as a binary classification problem. However, the selection of negative samples is a key problem in this case. In this study, a novel negative sample selection strategy was designed for accessing high-quality negative samples. Such strategy applied the random walk with restart (RWR) algorithm on a chemical-chemical interaction network to select pairs of drugs and side effects, such that drugs were less likely to have corresponding side effects, as negative samples. Through several tests with a fixed feature extraction scheme and different machine-learning algorithms, models with selected negative samples produced high performance. The best model even yielded nearly perfect performance. These models had much higher performance than those without such strategy or with another selection strategy. Furthermore, it is not necessary to consider the balance of positive and negative samples under such a strategy.

## 1. Introduction

Drugs are always special products for the treatment of various diseases. However, a drug is also a double-edged sword; it can bring some unexpected negative effects, usually called side effects, when it produces therapeutic effects. Side effects are almost inevitable for all drugs. Determining the side effects of drugs as early as possible can decrease the risks both for patients and pharmaceutical companies. It is reported that side effects cause 100,000 deaths per year in the United States [1]. On the other hand, an unacceptable side effect is the major reason for the failure of drug development. Even some launched drugs (e.g., Rofecoxib) had to be withdrawn after their unacceptable side effects were discovered. Thus, it is urgent to design effective methods to determine the side effects of drugs. However, it takes a lot of time and is of high costs to ascertain the side effects of a given drug through clinical trials. With the development of computer science, lots of advanced computational methods have been proposed, which give abundant resources to build effective computational models in this regard.

In recent years, many computational methods have been developed for predicting side effects of drugs. Among these methods, several of them built an individual classifier for each side effect [1–5]. They always took the drugs having a given side effect as positive samples and other drugs as negative samples. Clearly, to determine all side effects of a given drug, a large number of classifiers should be performed. Considering the fact that plenty of drugs have multiple side effects, some methods deemed the problem of predicting drug side effects as a multilabel classification problem [6–11]. It is a good idea to build a uniform frame to predict side effects of given drugs. However, these models are always complex and have high computational complexity. Different from the above methods, other methods built regression models for the prediction of drug side effects [12, 13]. Recently, some studies proposed a uniform binary classification model for predicting drug side effects [14–17]. They deemed the pairs of drugs and side effects as samples. A pair containing a drug and a side effect such that the drug has this side effect was termed as a positive sample and other pairs as negative samples. Because there were lots of negative samples, if all negative samples are selected, it is quite difficult to set up an effective prediction model. In some studies, they randomly selected some of them to build the model. It is clear that the utility of these constructed models relied on the selection of negative samples. Random selection of negative samples is not a rigorous way because some potential positive samples that have not been validated may be selected. Furthermore, selecting how many negative samples is also an important problem. It is necessary to design a refined strategy for picking up negative samples that are true negative samples with extreme high probabilities.

In this study, we did some work for selecting negative samples. A refined negative sample selection strategy was proposed to select high-quality negative samples. To this end, a drug network was constructed according to the chemical-chemical interaction (CCI) information retrieved from STITCH [18, 19]. Then, the random walk with restart (RWR) algorithm [20] was applied on the network to access high-quality negative samples. Based on obtained negative samples and positive samples retrieved from SIDER [21], classification models incorporating certain classification algorithms can be built, in which each sample was encoded into five features used in Zhao et al.'s study [14]. Three classification algorithms, random forest (RF) [22], support vector machine (SVM) [23], and artificial neural network (ANN), were adopted in this study. Several tests were performed to evaluate classification models with different classification algorithms and different quality negative samples. The best model gave the almost perfect classification. Furthermore, the proportion of positive and negative samples was not a problem when our negative sample selection strategy was used.

## 2. Materials and Methods

### 2.1. Materials

Drugs and their side effects used in this study were the same as those in our previous study [14]. In fact, this information was obtained from the well-known public database, SIDER [21]. The raw information contained a total of 888 drugs and 1385 side effects. With the same data cleaning procedures, we excluded the side effects with less than six drugs and drugs whose properties mentioned in Drug Properties and Associations were not available. Finally, 841 drugs and 824 side effects were accessed. In this study, the pairs of drugs and side effects were termed as samples. The above-mentioned drugs and side effects can comprise 57,058 pairs of drugs and side effects, which were deemed as positive samples. For convenience, these samples constituted the dataset PDS.

### 2.2. Negative Sample Selection Strategy

In Materials, the dataset PDS containing the positive pairs of drugs and side effects was constructed according to the information in SIDER. To construct the classification model, negative samples were necessary. In our previous study [14], negative samples were produced by randomly pairing drugs and side effects. Here, a refined strategy was proposed, which can generate high-quality negative samples.

#### 2.2.1. Drug Network

It has been reported in several studies that interacting chemicals are more likely to share similar properties [24–29]. It is feasible to adopt such information for investigating drug side effects because side effect is one of the important properties of drugs. In this study, we used the information of CCI to construct a drug network.

The CCI information was retrieved from STITCH (http://stitch.embl.de/, version 4.0) [18, 19], an online public database collecting known and predicted interactions between chemicals and proteins. These interactions were obtained by the evidence derived from experiments, databases, and the literature. Thus, they can widely measure the associations between chemicals and proteins. Each CCI in STITCH is assigned five scores, titled by “similarity,” “experimental,” “database,” “textmining,” and “combined_score,” with a range between 1 and 999. In detail, the first four scores measure the associations of chemicals according to their structures, activities, reactions and cooccurrence in the literature, while the last one integrates all above scores. Clearly, the last score can widely and accurately evaluate the linkages between chemicals. Thus, we used such score to construct the drug network. For formulation, let us denote the “combined_score” of chemicals *c*_1_ and *c*_2_ as *Q*(*c*_1_, *c*_2_).

The constructed drug network took 841 drugs as nodes, and two drugs were adjacent if and only if they can comprise a CCI with a “combined_score” larger than zero. Furthermore, to indicate the different strength of edges, each edge with *d*_1_ and *d*_2_ as endpoints was assigned a weight that was defined as *Q*(*d*_1_, *d*_2_).

#### 2.2.2. Random Walk with Restart Algorithm

The RWR algorithm is a powerful and widely used network ranking algorithm [20, 27, 30–33]. In this algorithm, the walker randomly moves from a seed node set to other nodes in the network. When the algorithm stops, each node in the network receives a probability, which can be deemed as an important indicator representing the essential associations to seed nodes. Given a seed node set SN, the RWR algorithm first constructs a probability vector, denoted as *p*_0_, in which the probability for each node in SN is defined as 1/|*SN*|, while probabilities for other nodes are set to zero. The RWR algorithm repeatedly updates this probability vector until it becomes stable. Let *p*_*t*_ represent such probability vector after the *t*-th iteration has been executed. Then, the probability vector *p*_*t*+1_ is updated by the following equation:

(1) $$\begin{array}{c} p_{t+1}=\left(1-\lambda \right)A^{T}p_{t}+\lambda p_{0}, \end{array}$$

where *λ* was set to 0.8, as used in other studies [27, 32–34], in this study and *A* represents the columnwise normalized adjacency matrix of the network. When ‖*p*_*t*+1_ − *p*_*t*_‖_*L*_1__ < *θ*, the update procedure stops, and *p*_*t*+1_ is picked up as the output of the RWR algorithm. In this study, *θ* was set to 10^‐6^.

The refined negative sample selection strategy is based on the above-mentioned drug network and RWR algorithm. For each drug side effect, we picked up the drugs owning such side effect as seed nodes of the RWR algorithm. Then, the RWR algorithm was applied on the drug network. When the RWR algorithm stopped, each node in the network was assigned a probability. It is clear that a node (drug) with a high probability had a strong association with seed nodes, thereby inferring that such node had a high probability of owning the side effect. On the contrary, nodes (drugs) with low probabilities were less likely to own the side effect. Given a threshold *ε* of the probability, drugs receiving the probabilities less than *ε* can be extracted, and they were paired with the side effect as the candidate negative samples. After considering all side effects, a negative sample set, denoted by NDS, was built by collecting all candidate negative samples for each side effect. This set was combined with PDS to constitute the training dataset.

### 2.3. Drug Properties and Associations

To encode each pair of drugs and side effects, we employed five drug properties, which were also used in our previous study [14]. Based on each property, a score evaluating the associations between two drugs can be obtained. Here, a brief description is given. The detailed description can be found in our previous study [14].

#### 2.3.1. Drug Association in Fingerprint

A drug can be represented by a SMILES (simplifying the molecular linear input specification) string [35], from which its fingerprints (ECFP_4) were extracted via RDKit [36]. Then, the Tanimoto coefficient is adopted to quantify the association between two drugs based on their fingerprints. For formulation, the thus-obtained association between drugs *d*_1_ and *d*_2_ is denoted by *W*^*f*^(*d*_1_, *d*_2_).

#### 2.3.2. Drug Association in Structure

Apart from the SMILES strings to represent drugs, drugs can also be represented by a graph [37]. Then, the association between two drugs can be assessed according to the sizes of two graphs and their maximum common subgraph. The online tool “SIMCOMP” in KEGG adopts such scheme to evaluate the associations of drugs [38]. The score between *d*_1_ and *d*_2_ obtained by “SIMCOMP” is denoted by *W*^*s*^(*d*_1_, *d*_2_).

#### 2.3.3. Drug Association in ATC Code

In the Anatomical Therapeutic Chemical (ATC) classification system, each drug is assigned one or more five-level ATC codes. According to the ATC codes of two drugs, their associations can be quantified. Detailed descriptions can be found in [14]. *W*^*c*^(*d*_1_, *d*_2_) is used to represent the associations of drugs in terms of their ATC codes.

#### 2.3.4. Drug Literature Association

The drug association can further be assessed by text-mining methods. Here, we adopted such association reported in STITCH [18, 19]. For drugs *d*_1_ and *d*_2_, their association is denoted by *W*^*l*^(*d*_1_, *d*_2_).

#### 2.3.5. Drug Association in Target Protein

A drug has one or more target proteins. This information can be represented by a 0-1 vector. Then, the association of two drugs can be quantified by the direction cosine of corresponding vectors. Let us denote such association between *d*_1_ and *d*_2_ by *W*^*t*^(*d*_1_, *d*_2_).

### 2.4. Feature Construction

Based on the five types of drug associations mentioned in Drug Properties and Associations, we used the “similarity” concept to extract features for each sample. For each type of drug association, one feature was extracted. Here, we gave a procedure for extracting one feature from the drug association in the fingerprint. Others can be obtained in a similar way.

For a sample containing a drug *d* and side effect *s*, let *S* be a drug set consisting of drugs owning side effect *s*. The feature derived from the drug association in the fingerprint for such sample was defined as

(2) $$\begin{array}{c} Q^{f}\left(d,s\right)=\max\left\{W^{f}\left(d,d^{\prime }\right)\;|\;d^{\prime }\in S-\left\{d\right\}\right\}, \end{array}$$

where *W*^*f*^(*d*, *d*′) indicated the strengthen of the association between *d* and *d*′ according to their fingerprints (see Drug Properties and Associations for detail). Obviously, a high *Q*^*f*^(*d*, *s*) meant the drug *d* was highly related to drugs owning side effect *s*. Thus, it was more likely to have side effect *s*.

Finally, each sample can be represented by a 5-dimension vector.

### 2.5. Classification Algorithm

Selecting a proper classification algorithm is very important for constructing an efficient classification model. This study adopted three classic algorithms: RF [22], SVM [23], and ANN. To quickly implement these algorithms, three tools “RandomForest,” “SMO,” and “MultilayerPerceptron” in Weka [39] were employed. For convenience, these tools were executed with their default parameters. Although the performance of models can be improved if more proper parameters were tried for each of the above-mentioned algorithms, it is not the keynote of this study. In our study, we tried to prove that the quality of negative samples selected by the proposed negative sample selection strategy was high no matter which algorithm was chosen as the prediction engine.

### 2.6. Performance Measurement

This study modeled a binary classification model for the prediction of drug side effects. For a binary classification problem, several measurements can be calculated to evaluate the performance of the model. In this study, we used the following measurements: *Recall* (also known as Sensitivity (SN) and *true positive rate* (TPR)), *false positive rate* (FPR), *Specificity (SP), prediction accuracy* (ACC), *Matthews correlation coefficient* (MCC) [40], *Precision*, and *F*1‐*measure* [41]. Their formulations are as follows:

(3) $$\begin{array}{c} \text{Recall}=\text{SN}=\text{TPR}=\frac{\text{TP}}{\text{TP}+\text{FN}},\\ \text{FPR}=\frac{\text{FP}}{\text{FP}+\text{TN}},\\ \text{SP}=\frac{\text{TN}}{\text{TN}+\text{FP}},\\ \text{ACC}=\frac{\text{TP}+\text{TN}}{\text{TP}+\text{FN}+\text{FP}+\text{TN}},\\ \text{MCC}=\frac{\text{TP}\times \text{TN}-\text{FP}\times \text{FN}}{\sqrt{\left(\text{TN}+\text{FN}\right)\times \left(\text{TN}+\text{FP}\right)\times \left(\text{TP}+\text{FN}\right)\times \left(\text{TP}+\text{FP}\right)}},\\ \text{Precision}=\frac{\text{TP}}{\text{TP}+\text{FP}},\\ \text{F}1‐\text{measure}=\frac{2\times \text{Precision}\times \text{Recall}}{\text{Precision}+\text{Recall}}, \end{array}$$

where TP and TN represent true positive and true negative, respectively, while FP and FN indicate false positive and false negative, respectively.

Besides, to fully evaluate the performance of different classification models, we further employed a receiver operating characteristic (ROC) curve and a precision-recall (PR) curve. By setting several thresholds for predicting positive samples, a series of TPRs, FPRs, and Precisions can be obtained. The ROC curve takes the TPR as the *y*-axis and FPR as the *x*-axis. Likewise, the PR curve sets the Precision as the *y*-axis and Recall as the *x*-axis. The areas under these two curves, called AUROC and AUPR, respectively, can be further calculated to assess the performance of the model. Clearly, a high AUROC or AUPR indicates high performance.

## 3. Results and Discussion

**Figure 1:**
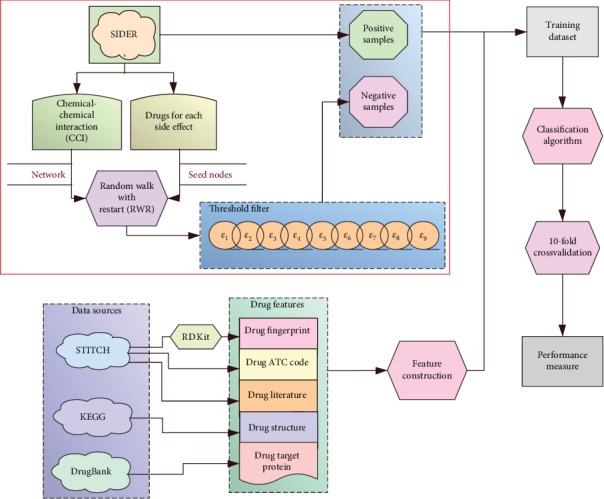
Entire procedures of the construction of classification models with a refined negative sample selection strategy.

In this study, a binary classification model incorporating a refined negative sample selection strategy was proposed to predict drug side effects. The whole procedures are illustrated in Figure 1. This section gave detailed testing results of different models and made further analysis.

### 3.1. Negative Samples with Different Thresholds of Probability

The negative sample selection strategy applied the RWR algorithm to the drug network and extracted negative samples according to the threshold *ε* of the probability. We tried nine values of *ε* to construct nine different NDSs. The numbers of negative samples under different values of *ε* are listed in Table 1. It can be observed that the numbers of negative samples followed an increasing trend with the increasing of *ε*.

**Figure 2:**
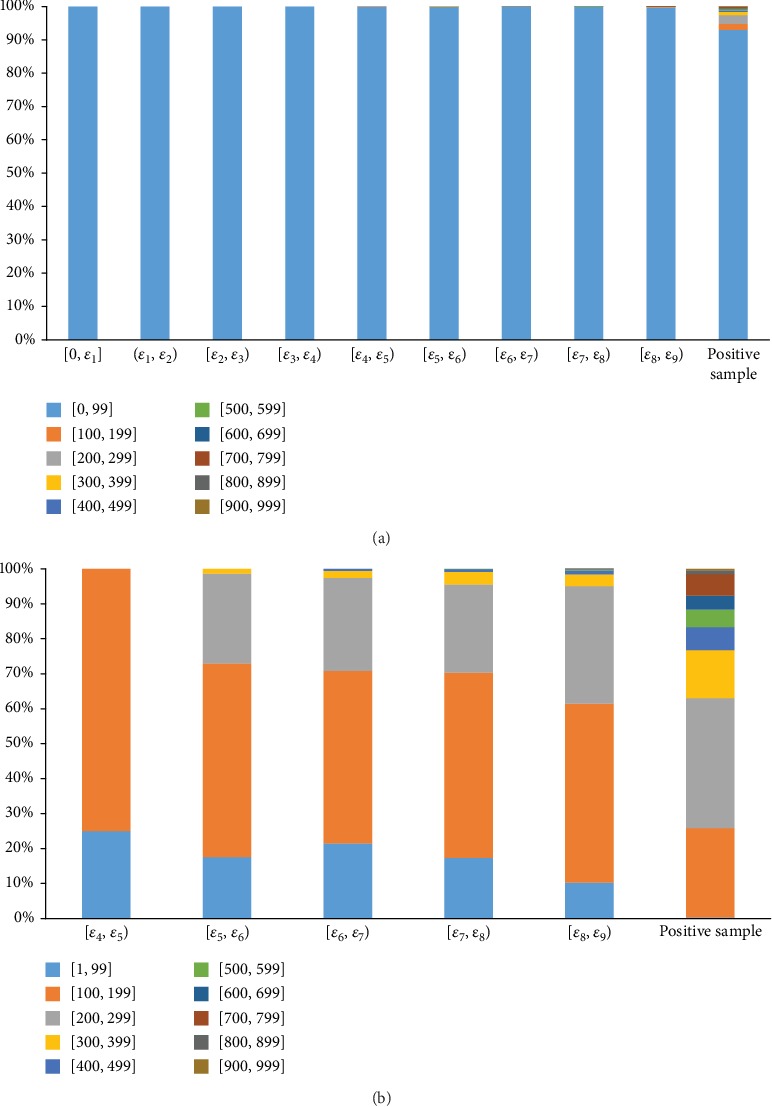
Distribution of “combined_score” of drugs and drugs sharing the side effects for negative samples in nine parts and positive samples. (a) Zero scores were included; (b) zero scores were not included.

According to the principle of the RWR algorithm, it can be inferred that negative samples obtained by small *ε* were of high quality. To confirm this, based on nine thresholds listed in Table 1, we divided 333,797 negative samples selected by setting the threshold *ε* = *ε*_9_ into nine parts ([0, *ε*_1_], (*ε*_1_, *ε*_2_), [*ε*_2_, *ε*_3_), [*ε*_3_, *ε*_4_), [*ε*_4_, *ε*_5_), [*ε*_5_, *ε*_6_), [*ε*_6_, *ε*_7_), [*ε*_7_, *ε*_8_), and [*ε*_8_, *ε*_9_)). Then, for each negative sample with the drug *d* and side effect *s*, the “combined_score” between *d* and drugs owing side effect *s* was extracted. For each part, we counted the proportions of such scores in ten intervals from 0 and 999, which are illustrated in Figure 2, where Figure 2(a) considers zero scores, whereas Figure 2(b) excludes these scores. It can be observed from Figure 2 that all scores were zeros for the first four parts, indicating that the drug in each of these samples had no direct links to drugs owning the side effect in the same sample. It is suggested that this drug shared such side effect with a quite low probability. For the following five parts, they contained more and more high scores, implying that in some samples, drugs had direct links to those sharing the side effects and these links became stronger. Thus, it can be deduced that drugs had the side effects with higher probabilities than the samples in the first four parts. In Figure 2, we also counted the scores of positive samples. Score distributions of some of the first parts were quite different from those of the positive samples, and with increase of the probability, the distribution became more and more similar to that of the positive samples. This suggested that with the increase of the part index, samples became more and more similar to positive samples. With the above analysis, it can be partly concluded that the quality of samples decreased with the increase of the part index. Thus, with the increase of the threshold, quality of selected negative samples became worse and worse because more and more negative samples with low quality were poured in. Especially when *ε* = 0, 128,220 negative samples were of the highest quality.

### 3.2. Performance of the Models with the Highest Quality Negative Samples

**Figure 3:**
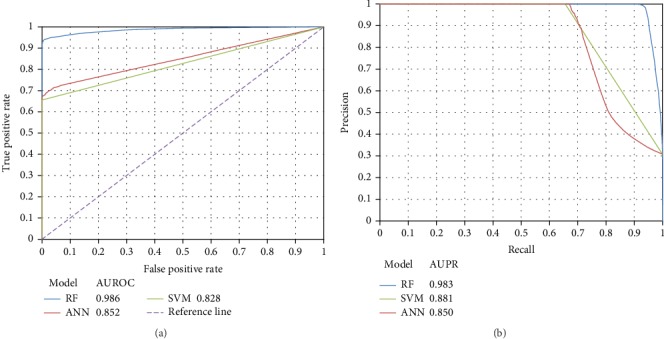
The ROC curves and PR curves of three models with the highest quality negative samples. (a) The ROC curves; (b) the PR curves.

As mentioned in Negative Samples with Different Thresholds of Probability, 128,220 negative samples were obtained when *ε* = 0. These samples were deemed to be of the highest quality. Based on them and three classification algorithms: RF, SVM and ANN, three models, named as RF, SVM, and ANN models, respectively, were built. Then, tenfold crossvalidation [42–45] was adopted to evaluate their performance. Six measurements, SN, SP, ACC, MCC, Precision, and F1‐measure, mentioned in Performance Measurement, were calculated and are listed in Table 2. It can be observed that the RF model yielded the best performance. The MCC, ACC, and F1‐measure obtained by the RF model were 0.943, 0.975, and 0.959, respectively. As for the SVM and ANN models, they produced perfect SPs and Precisions; however, their SNs were much lower, inducing much lower ACCs, MCCs, and F1‐measures. In addition, we plotted the ROC curves and PR curves of these models, as shown in Figure 3. Clearly, the ROC curve of the RF model was always above those of the SVM and ANN models. It was also true for the PR curve. The AUROC and AUPR of the RF model was 0.986 and 0.983, respectively, indicating the high utility of the RF model. The AUROCs and AUPRs of the other two models were at least 10% lower than those of the RF model. Therefore, it is suggested to select RF as the classification algorithm for building the classification model.

### 3.3. Performance of the Models with Different Quality Negative Samples

**Figure 4:**
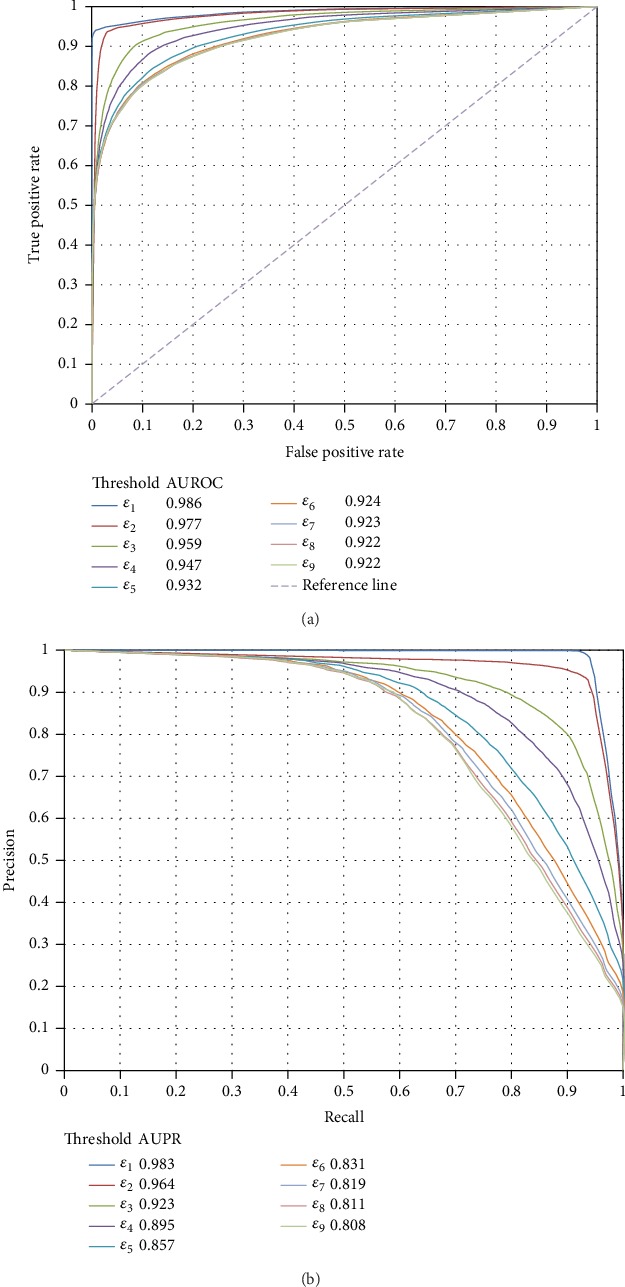
The ROC curves and PR curves of the RF models with different quality negative samples. (a) The ROC curves; (b) the PR curves.

Given different thresholds of the probability, we can obtain different negative samples and construct different models. As listed in Table 1, nine thresholds were tried in this study. For RF, we constructed nine RF models. These models were evaluated by tenfold crossvalidation. The results are listed in Table 3 from which we can see that the MCCs followed a general decreasing trend with the increase of the threshold. ACC and F1‐measure also followed such trend. It is reasonable because when the threshold increased, more and more negative samples were added and their quality became poorer. It can also be concluded from Table 3 that when the thresholds were small, the performance of the RF model followed a sharp decreasing trend with the increase of the threshold, while this trend became alleviative when the thresholds were large. With the increase of the threshold, the added negative samples were poor enough which cannot influence the performance of the model a lot. Besides, we also plotted the ROC curves and PR curves yielded by these RF models, as shown in Figure 4. The AUROCs and AUPRs followed the similar trend of ACCs, MCCs, and F1‐measures. Thus, it is better to use a small threshold for determining negative samples.

To prove that the above results were not special for RF, we also did the same tests for SVM and ANN. The predicted results are provided in Table S1 and S2. The ROC curves and PR curves are available in Figure S1 and S2. All results were almost identical to those of the RF models, indicating that with the increase of the threshold, the quality of negative samples became poorer. It is suggested to extract negative samples with a small threshold.

### 3.4. Analysis of the Models on Balanced and Imbalanced Datasets

For the problem investigated in this study, it is a dilemma to determine the number of negative samples. Based on our negative sample selection strategy, it is not a problem. The negative samples can be determined by a proper threshold, which suggests choosing a small threshold.

It can be seen that the above-constructed models all used much more negative samples than positive samples (Table 1). For example, when *ε* = 0, the negative samples were more than twice the positive samples. With the increase of *ε*, the negative samples became more and more. Thus, the above-constructed models were all based on imbalanced datasets. This section proved that when the threshold was given, the proportion of positive and negative samples cannot be considered. To this end, we did the following tests.

**Figure 5:**
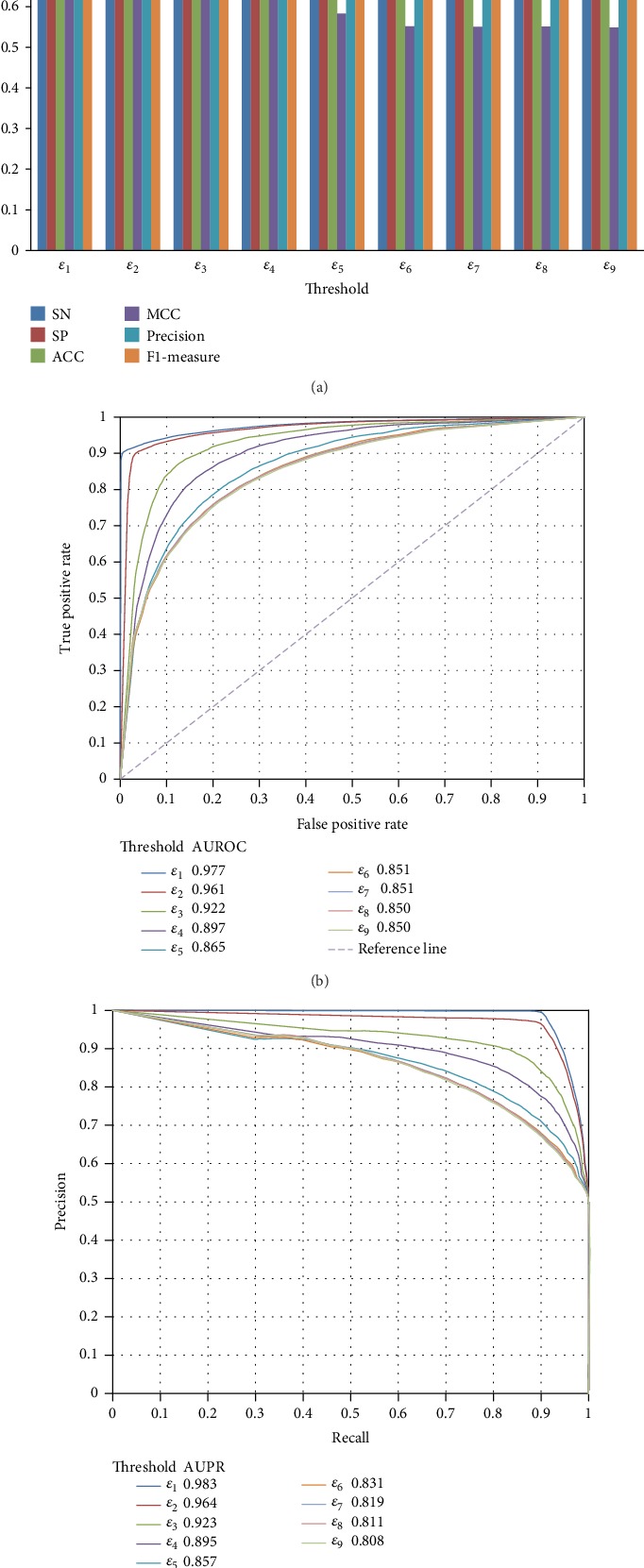
The performance of the RF models on balanced datasets, in which negative samples, as many as positive samples, are randomly selected under different thresholds. (a) Six measurements; (b) the ROC curves; (c) the PR curves.

**Figure 6:**
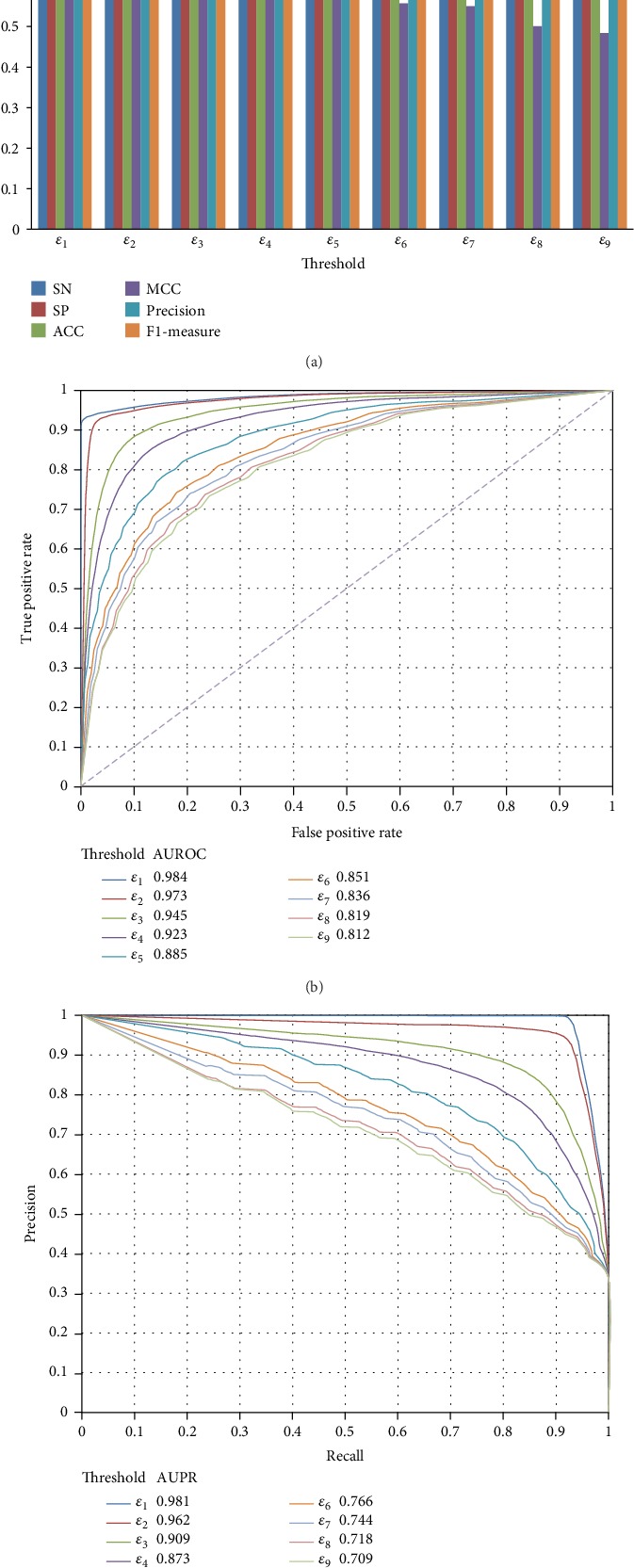
The performance of the RF models on imbalanced datasets, in which negative samples, twice as many as positive samples, are randomly selected under different thresholds. (a) Six measurements; (b) the ROC curves; (c) the PR curves.

For a given threshold *ε*, we can obtain several negative samples. Among them, we randomly selected negative samples, which were as many as positive samples. These selected negative samples were combined with positive samples to construct a balanced dataset. Because the selection of negative samples may influence the results, we constructed four additional datasets in the same way. Thus, five balanced datasets, denoted by BD_1_^*ε*^_,_…, BD_5_^*ε*^, were constructed. Furthermore, we constructed five imbalanced datasets in a similar way. These datasets contained negative samples twice as many as positive samples. These imbalanced datasets were denoted by IBD_1_^*ε*^,…, IBD_5_^*ε*^. A RF model was built based on each of the above-mentioned datasets and evaluated by tenfold crossvalidation. The performance of these RF models on balanced datasets is shown in Figure 5. It can be observed that with the increase of the threshold, the performance of RF models decreased, which conformed to the results in Performance of the Models with Different Quality Negative Samples. Furthermore, the performance of the RF models on imbalanced datasets is illustrated in Figure 6, giving the same conclusion. In addition, SVM and ANN models were also constructed on the above-mentioned balanced and imbalanced datasets. Their performance, evaluated by tenfold crossvalidation, is shown in Figure S3-S6. The same conclusion can be arrived at; that is, the performance of the models decreased when the threshold increased.

**Figure 7:**
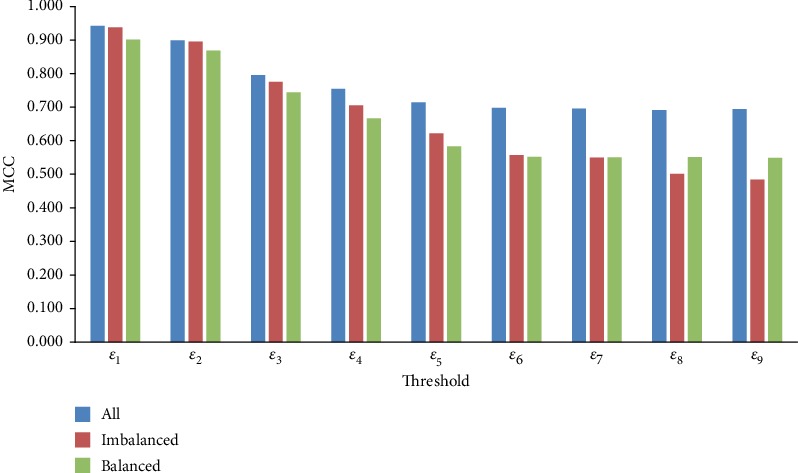
The MCCs yielded by the RF models on three types of datasets. “All” means that all negative samples under the given threshold are selected; “Imbalanced” indicates that negative samples, twice as many as positive samples, under the given threshold are randomly selected; and “Balanced” indicates that negative samples, as many as positive samples, under the given threshold are randomly selected.

Given a threshold *ε*, three types of datasets were constructed. The first one contained all negative samples; the second one, imbalanced datasets, containing negative samples twice as many as positive samples; and the last one, balanced datasets, containing negative samples as many as positive samples. As shown in Table 1, the first type of dataset had the highest imbalanced degree, followed by the second and third ones. Here, we investigated the performance of RF models on these three types of datasets under different thresholds of the probability. The MCCs are illustrated in Figure 7. It is interesting that given a threshold, the model on the first type of datasets always provided the best performance although it contained much more negative samples than the other two types of datasets. The reason may be that negative samples under a certain threshold were quite similar for the RF model; thus, employing more negative samples can improve the performance. For the two other types of datasets, when the threshold was smaller than or equal to *ε*_6_, RF models on imbalanced datasets were superior to those on balanced datasets, while it became contrary when the threshold was larger than *ε*_6_. It is indicated that there existed a critical value to control the performance of the RF model on balanced and imbalanced datasets. Furthermore, we investigated the performance of the SVM and ANN models on three types of datasets. Obtained MCCs are illustrated in Figure S7 and S8. For the SVM and ANN models, their performance was not always best on the first type of datasets when the threshold was fixed. However, when the threshold was small (smaller than *ε*_3_), the first type of dataset still yielded the best performance. For the second (imbalanced) and third (balanced) types of datasets, similar phenomena occurred. The only difference was the different critical values for SVM and ANN.

All in all, when we used threshold *ε*, which was suggested to be small in Performance of the Models with Different Quality Negative Samples, to determine the candidate negative samples, it is better to pick up all these candidates to construct the model, and it was not necessary to consider the proportion of positive and negative samples in this case.

### 3.5. Comparison of the Model without Negative Sample Selection

In this study, we proposed a refined negative sample selection strategy to extract high-quality negative samples. When using the highest quality negative samples, the RF model produced the best performance, listed in Table 2. If such strategy was not adopted, we randomly selected negative samples that were as many as positive samples to construct the RF model, which was identical to that in our previous study [14]. The predicted results yielded by the tenfold crossvalidation are listed in Table 4. It is easy to see that our model was much superior to the previous model. Each measurement was improved more than 10%. In detail, the ACC, MCC, and F1‐measure improved about 20%, 40%, and 18%, respectively. Thus, the proposed negative sample selection strategy can sharply improve the utility of the model. Furthermore, we also did the same comparisons for the SVM and ANN models. Predicted results are also listed in Table 4. The same conclusion can be obtained.

### 3.6. Comparison of the Model with Another Negative Sample Selection Strategy

In [46], another negative sample selection strategy, namely, finding reliable negative samples (FIRE), was proposed to improve the model for predicting protein-RNA interactions. This strategy was employed in this study to compare with the proposed strategy. We termed drugs as proteins and side effects as RNAs in FIRE. Furthermore, the “combined_score” between drugs was deemed as the protein-protein similarity score in FIRE. According to FIRE, each pair of drug and side effect that was not a positive sample was assigned a score. It was claimed in [46] that samples with low scores were of high quality. Thus, we picked up the pairs of drugs and side effects with zero scores as negative samples for making comparison, obtaining 355,634 negative samples. The negative samples with the highest quality (using threshold *ε*_1_) filtered by our strategy were selected to make comparison. Several RF models were constructed on these two different negative sample sets and the same positive samples.

**Figure 8:**
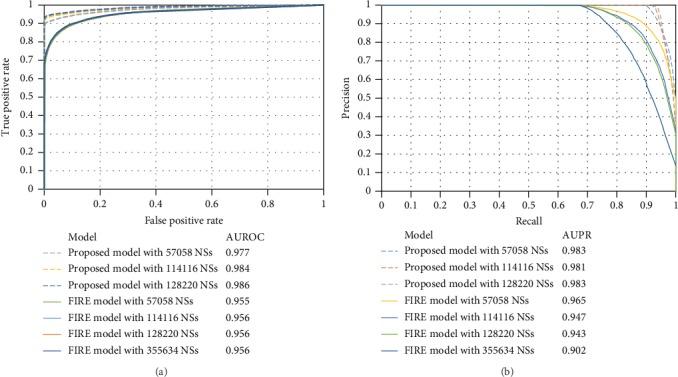
ROC and PR curves of models with different negative samples obtained by two different negative sample selection strategies. (a) The ROC curves; (b) the PR curves. The proposed model is constructed with negative samples obtained by the proposed strategy, whereas the FIRE model is built with negative samples obtained by FIRE. NS: negative sample.

First, we compared the RF models with balanced positive and negative samples; that is, 57,058 negative samples were randomly selected from two negative sample sets, which were combined with the positive samples to construct RF models. Tenfold crossvalidation results are listed in Table 5. Clearly, the RF model obtained by the proposed strategy (called the proposed model in the following text for convenience) was superior to that obtained by FIRE (called the FIRE model in the following text for convenience). The MCC was 10.7% higher. Furthermore, we also did the ROC and PR curve analyses, which are shown in Figure 8. Clearly, the ROC and PR curves of the proposed model were always above the corresponding curves of the FIRE model. The AUROC and AUPR were 2.2% and 1.8% higher, respectively. Thus, the proposed model was better than the FIRE model. Second, we compared the RF models with imbalanced positive and negative samples. In this case, negative samples were twice as many as positive samples. According to the results listed in Table 5 and Figure 8, the proposed model was also superior to the FIRE model. Third, the RF models with all samples in two negative sample sets were constructed to make comparison. The results are also listed in Table 5 and Figure 8. The MCC of the FIRE model was 0.812, which was 13.1% lower than that of the proposed model. As for AUROC and AUPR, they were 3% and 8.1% lower than those of the proposed model, respectively. It was also indicated that the proposed model was better than the FIRE model. Finally, considering the fact that negative samples selected by FIRE were much more than those filtered by the proposed strategy, we randomly selected 128,220 samples from the negative samples obtained by FIRE and used them to construct the RF model. The tenfold crossvalidation results are also listed in Table 5 and Figure 8. Clearly, such model was inferior to the proposed model with the same number of negative samples. According to the above arguments, the proposed models were superior to the FIRE models, proving that the proposed negative sample selection strategy can screen out negative samples with higher quality than FIRE.

**Figure 9:**
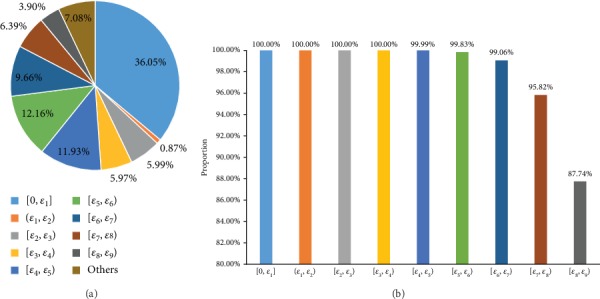
Breakdown of the negative samples selected by FIRE. (a) Distribution of such negative samples on nine parts of negative samples obtained by the proposed strategy. (b) Proportions of negative samples selected by FIRE in each part of the negative samples obtained by the proposed strategy.

From the above arguments, negative samples selected by the proposed strategy were of higher quality than those filtered by FIRE. Here, we provided an investigation to explain the reason.  Figure 9(a) shows the distribution of 355,634 negative samples selected by FIRE on nine parts of negative samples mentioned in Negative Samples with Different Thresholds of Probability. Each of the nine parts contained several such negative samples. For example, the first part contained 36.05% such negative samples and the second part contained 0.87% such negative samples. This result suggested that our strategy can classify negative samples generated by FIRE into different parts, which contained negative samples with different quality. Furthermore, as shown in Figure 9(b), all negative samples generated by the proposed strategy with threshold *ε*_4_ were selected by FIRE, and this proportion decreased with the increase of the threshold. Most negative samples in each part (more than 87%) were also selected by FIRE. Thus, our strategy improved the evaluation scheme on negative samples and gave a more refined partition on negative samples. FIRE evaluated the quality of negative samples by only considering the direct links between drugs. If the distances between one drug and drugs sharing one side effect were all larger than one, such pair of drug and side effect was assigned a zero score and deemed as a negative sample with the highest quality by FIRE. FIRE did not consider the factor of distance. In fact, such pairs can be further classified. Pairs with long distances were clearly more likely to be actual negative samples. For the proposed strategy, it adopted the RWR algorithm to evaluate the quality of negative samples. Generally, pairs with long distances would be assigned low probabilities. Therefore, we can further classify negative samples selected by FIRE into many parts by setting different thresholds on the probability. However, FIRE cannot divide them because their scores were all zeros. All these induced the phenomenon shown in Figure 9, and it was the main reason why our strategy was better than FIRE.

## 4. Conclusions

This study proposed a novel negative sample selection strategy for the prediction of drug side effects. Under a small threshold, the negative samples are of high quality, indicating that it is not necessary to consider the balance of positive and negative samples. It is hopeful this strategy can give useful help for determining novel side effects of given drugs and new insights for dealing with similar biological and medicine problems.

## Figures and Tables

**Table 1:** Numbers of negative samples under different thresholds of the probability.

Tag of threshold	Threshold	Number of negative samples	Times of positive samples
*ε*_1_	0	128,220	2.25
*ε*_2_	5 × 10^−7^	131,301	2.30
*ε*_3_	5 × 10^−6^	152,596	2.67
*ε*_4_	1 × 10^−5^	173,822	3.05
*ε*_5_	2 × 10^−5^	216,256	3.79
*ε*_6_	3 × 10^−5^	259,566	4.55
*ε*_7_	4 × 10^−5^	294,260	5.16
*ε*_8_	5 × 10^−5^	317,971	5.57
*ε*_9_	6 × 10^−5^	333,797	5.85

**Table 2:** The performance of three models with the highest quality negative samples.

Model	SN	SP	ACC	MCC	Precision	F1‐measure
RF model	0.923	0.999	0.975	0.943	0.997	0.959
SVM model	0.656	1.000	0.894	0.754	1.000	0.792
ANN model	0.670	1.000	0.898	0.764	1.000	0.802

**Table 3:** The performance of the RF models with different quality negative samples.

Threshold	SN	SP	ACC	MCC	Precision	F1‐measure
*ε*_1_	0.923	0.999	0.975	0.943	0.997	0.959
*ε*_2_	0.910	0.978	0.958	0.899	0.948	0.929
*ε*_3_	0.816	0.960	0.921	0.796	0.883	0.849
*ε*_4_	0.751	0.964	0.912	0.754	0.873	0.808
*ε*_5_	0.668	0.975	0.911	0.715	0.877	0.758
*ε*_6_	0.622	0.982	0.917	0.698	0.884	0.730
*ε*_7_	0.605	0.986	0.924	0.695	0.890	0.720
*ε*_8_	0.594	0.987	0.927	0.691	0.891	0.713
*ε*_9_	0.588	0.989	0.930	0.694	0.901	0.712

**Table 4:** Comparison of the models with or without negative sample selection strategy.

Model	Negative sample selection strategy	SN	SP	ACC	MCC	Precision	F1‐measure
RF model	√(with *ε*_1_)	0.923	0.999	0.975	0.943	0.997	0.959
	×	0.791	0.759	0.775	0.550	0.766	0.778
SVM model	√(with *ε*_1_)	0.656	1.000	0.894	0.754	1.000	0.792
	×	0.585	0.715	0.650	0.302	0.672	0.625
ANN model	√(with *ε*_1_)	0.670	1.000	0.898	0.764	1.000	0.802
	×	0.631	0.695	0.663	0.332	0.682	0.650

**Table 5:** Comparison of RF models with two different negative sample selection strategies.

Negative sample selection strategy	Number of selected negative samples	SN	SP	ACC	MCC	Precision	F1‐measure
Proposed strategy	57,058	0.904	0.994	0.949	0.901	0.993	0.946
	114,116	0.919	0.999	0.972	0.938	0.997	0.956
	128,220	0.923	0.999	0.975	0.943	0.997	0.959
	57,058	0.887	0.907	0.897	0.794	0.905	0.896
FIRE	114,116	0.844	0.954	0.917	0.811	0.901	0.872
	128,220	0.832	0.961	0.921	0.812	0.904	0.867
	355,634	0.745	0.992	0.957	0.812	0.934	0.829

## Data Availability

The original data used to support the findings of this study are available at SIDER and in supplementary information files.

## Supplementary Materials

Supplementary Materials Table S1: the performance of the SVM models with different quality negative samples. Table S2: the performance of the ANN models with different quality negative samples. Figure S1: the ROC curves and PR curves of the SVM models with different quality negative samples. (A) The ROC curves; (B) the PR curves. Figure S2: the ROC curves and PR curves of the ANN models with different quality negative samples. (A) The ROC curves; (B) the PR curves. Figure S3: the performance of the SVM models on balanced datasets, in which negative samples, as many as positive samples, are randomly selected under different thresholds. (A) Six measurements; (B) the ROC curves; (C) the PR curves. Figure S4: the performance of the SVM models on imbalanced datasets, in which negative samples, twice as many as positive samples, are randomly selected under different thresholds. (A) Six measurements; (B) the ROC curves; (C) the PR curves. Figure S5: the performance of the ANN models on balanced datasets, in which negative samples, as many as positive samples, are randomly selected under different thresholds. (A) Six measurements; (B) the ROC curves; (C) the PR curves. Figure S6: the performance of the ANN models on imbalanced datasets, in which negative samples, twice as many as positive samples, are randomly selected under different thresholds. (A) Six measurements; (B) the ROC curves; (C) the PR curves. Figure S7: the MCCs yielded by the SVM models on three types of datasets. “All” means that all negative samples under the given threshold are selected; “Imbalanced” indicates that negative samples, twice as many as positive samples, under the given threshold are randomly selected; and “Balanced” indicates that negative samples, as many as positive samples, under the given threshold are randomly selected. Figure S8: the MCCs yielded by the ANN models on three types of datasets. “All” means that all negative samples under the given threshold are selected; “Imbalanced” indicates that negative samples, twice as many as positive samples, under the given threshold are randomly selected; and “Balanced” indicates that negative samples, as many as positive samples, under the given threshold are randomly selected.
