# Supplementary material for: Prediction of Drug Side Effects with a Refined Negative Sample Selection Strategy
Source: Comput Math Methods Med. 2020 May 9;2020:1573543. doi: 10.1155/2020/1573543 (PMC7232712; doi:10.1155/2020/1573543)
Supplement: Supplementary Materials — Table S1: the performance of the SVM models with different quality negative samples. Table S2: the performance of the ANN models with different quality negative samples. Figure S1: the ROC curves and PR curves of the SVM models with different quality negative samples. (A) The ROC curves; (B) the PR curves. Figure S2: the ROC curves and PR curves of the ANN models with different quality negative samples. (A) The ROC curves; (B) the PR curves. Figure S3: the performance of the SVM models on balanced datasets, in which negative samples, as many as positive samples, are randomly selected under different thresholds. (A) Six measurements; (B) the ROC curves; (C) the PR curves. Figure S4: the performance of the SVM models on imbalanced datasets, in which negative samples, twice as many as positive samples, are randomly selected under different thresholds. (A) Six measurements; (B) the ROC curves; (C) the PR curves. Figure S5: the performance of the ANN models on balanced datasets, in which negative samples, as many as positive samples, are randomly selected under different thresholds. (A) Six measurements; (B) the ROC curves; (C) the PR curves. Figure S6: the performance of the ANN models on imbalanced datasets, in which negative samples, twice as many as positive samples, are randomly selected under different thresholds. (A) Six measurements; (B) the ROC curves; (C) the PR curves. Figure S7: the MCCs yielded by the SVM models on three types of datasets. “All” means that all negative samples under the given threshold are selected; “Imbalanced” indicates that negative samples, twice as many as positive samples, under the given threshold are randomly selected; and “Balanced” indicates that negative samples, as many as positive samples, under the given threshold are randomly selected. Figure S8: the MCCs yielded by the ANN models on three types of datasets. “All” means that all negative samples under the given threshold are selected; “Imbalanced” indicates that negative sampl [file 1573543.f1.pdf]

Table S1. The performance of the SVM models with different quality negative samples

Threshold	SN	SP	ACC	MCC	precision	F1-measure
ε_1	0.656	1.000	0.894	0.754	1.000	0.792
ε_2	0.651	0.985	0.884	0.721	0.950	0.773
ε_3	0.545	0.932	0.826	0.533	0.749	0.631
ε_4	0.526	0.872	0.787	0.411	0.575	0.549
ε_5	0.000	1.000	0.791	0.000	0.000	0.000
ε_6	0.000	1.000	0.820	0.000	0.000	0.000
ε_7	0.000	1.000	0.838	0.000	0.000	0.000
ε_8	0.000	1.000	0.848	0.000	0.000	0.000
ε_9	0.000	1.000	0.854	0.000	0.000	0.000

Table S2. The performance of the ANN models with different quality negative samples

**Threshold**	**SN**	**SP**	**ACC**	**MCC**	**precision**	**F1-measure**
ε_1	0.670	1.000	0.898	0.764	1.000	0.802
ε_2	0.680	0.982	0.891	0.737	0.943	0.791
ε_3	0.641	0.922	0.846	0.595	0.756	0.693
ε_4	0.506	0.906	0.807	0.452	0.652	0.559
ε_5	0.198	0.969	0.808	0.273	0.634	0.298
ε_6	0.084	0.987	0.824	0.145	0.411	0.140
ε_7	0.059	0.991	0.839	0.125	0.438	0.104
ε_8	0.035	0.993	0.848	0.067	0.235	0.060
ε_9	0.000	1.000	0.854	0.000	0.000	0.000

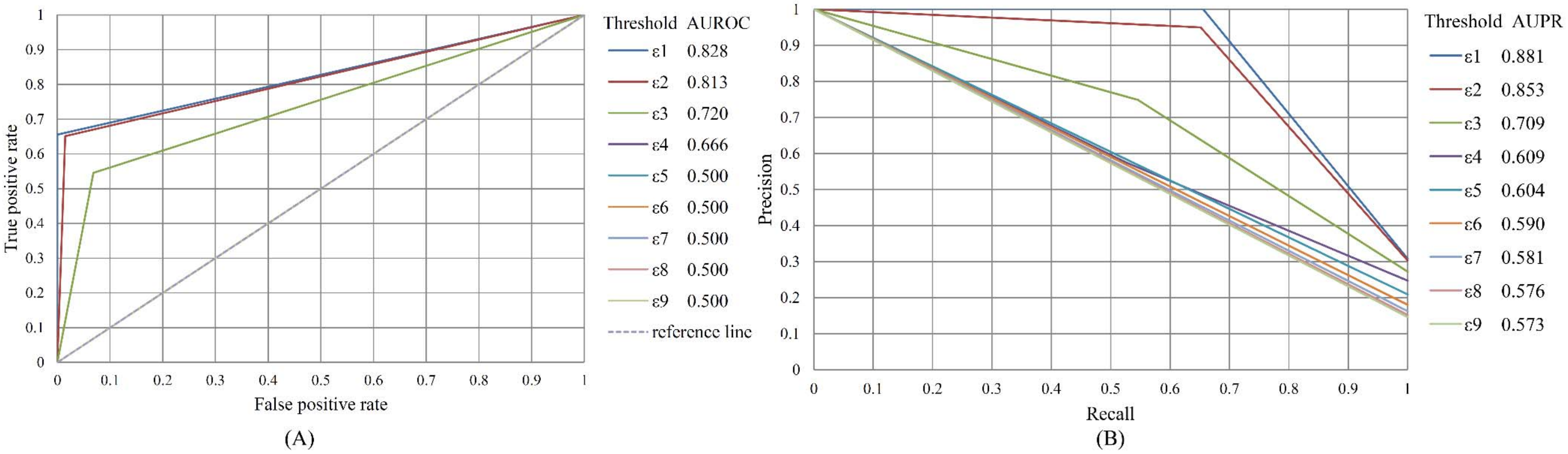

Figure S1. The ROC cures and PR curves of the SVM models with different quality negative samples. (A) The ROC curves; (B) The PR curves.

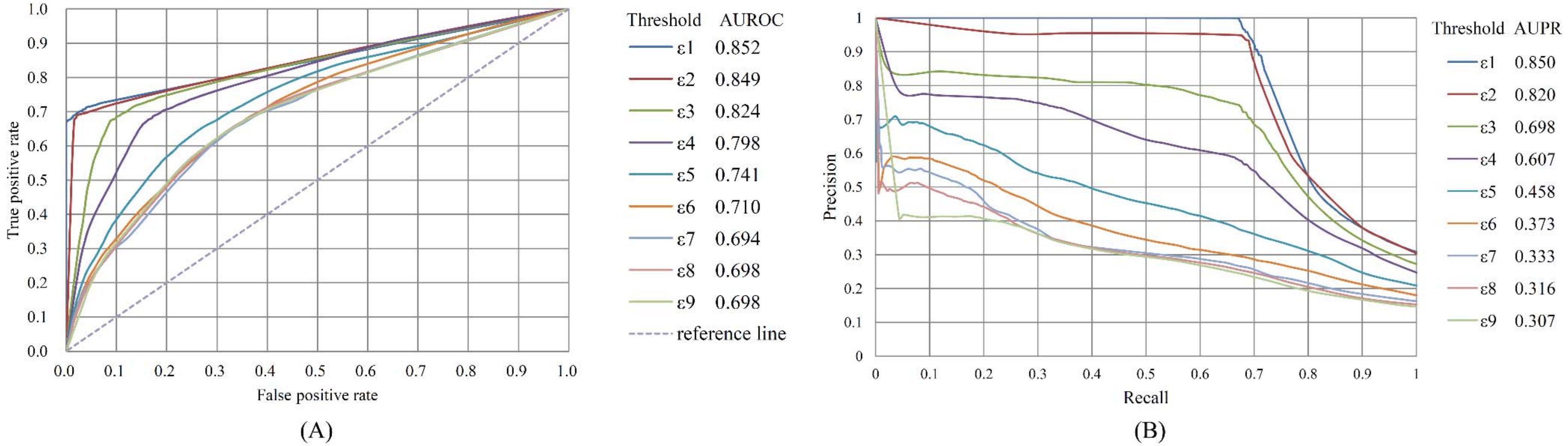

Figure S2. The ROC cures and PR curves of the ANN models with different quality negative samples. (A) The ROC curves; (B) The PR curves.

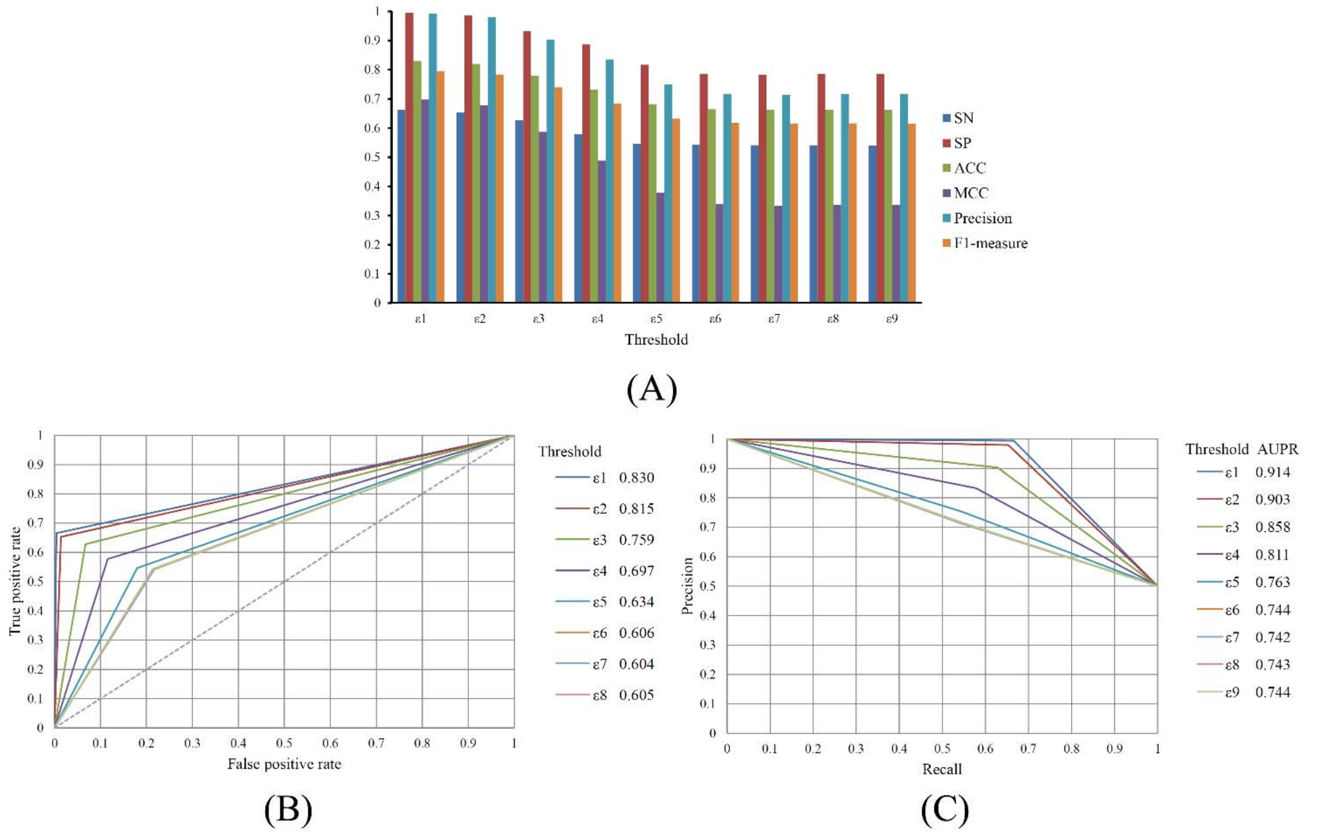

Figure S3. The performance of the SVM models on balanced datasets, in which negative samples, as many as positive samples, are randomly selected under different thresholds. (A) Six measurements; (B) The ROC curves; (C) The PR curves.

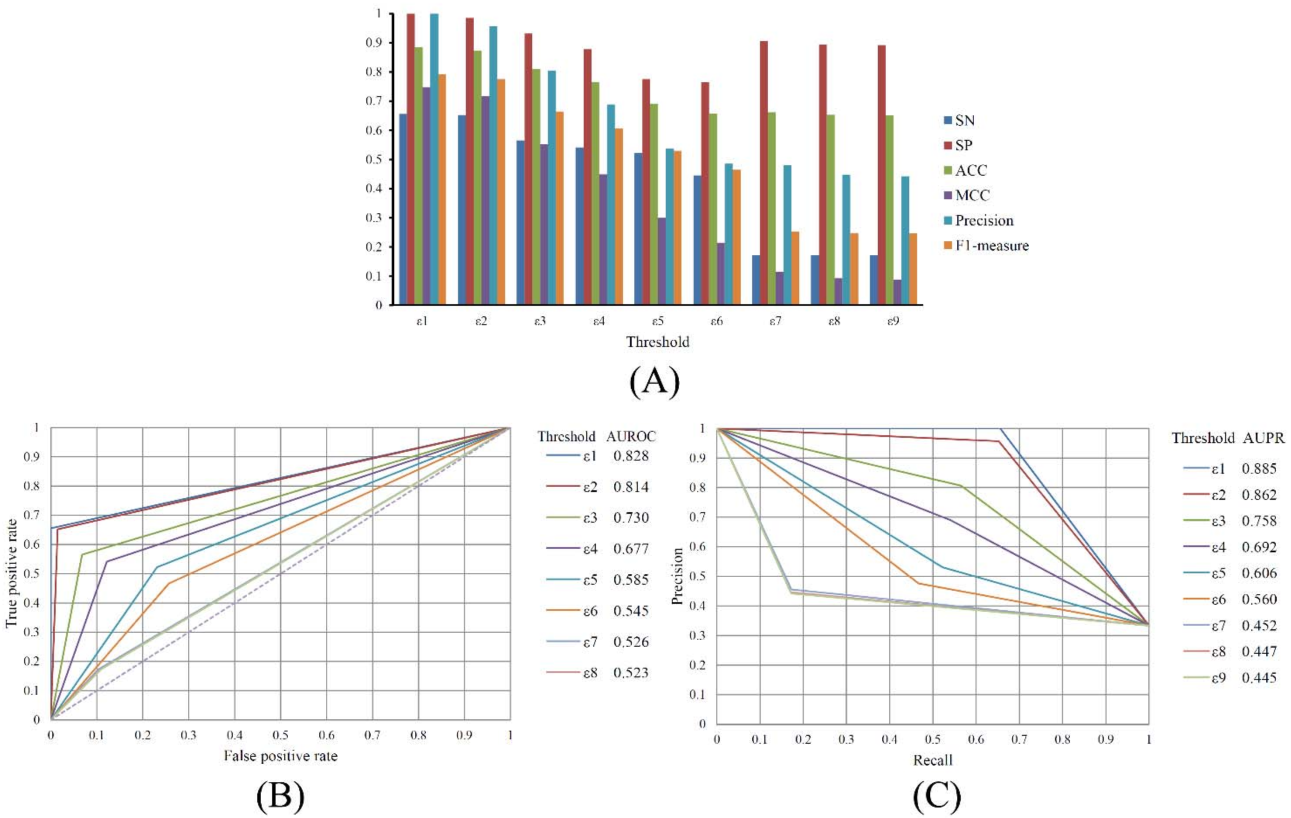

Figure S4. The performance of the SVM models on imbalanced datasets, in which negative samples, twice as many as positive samples, are randomly selected under different thresholds. (A) Six measurements; (B) The ROC curves; (C) The PR curves.

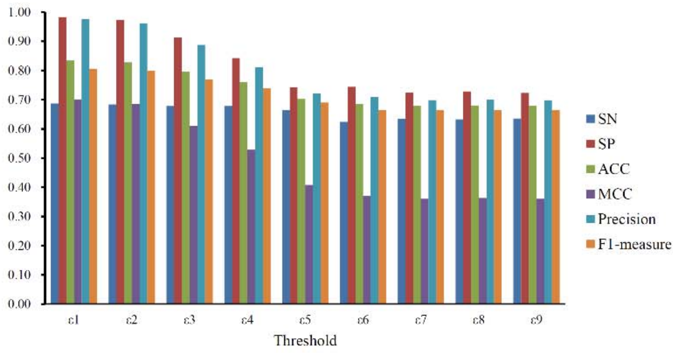

(A)

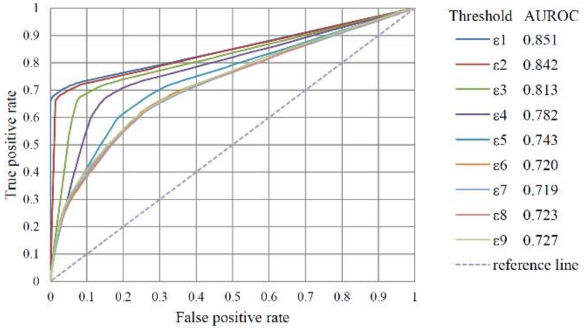

(B)

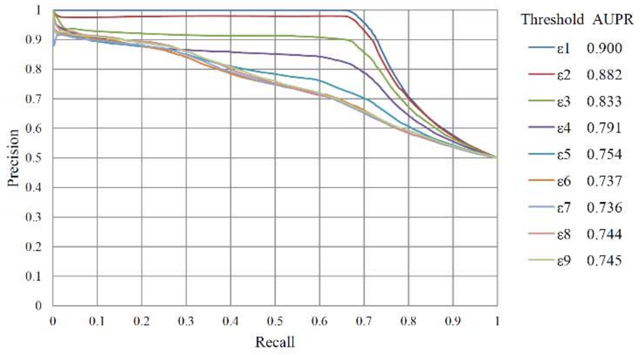

(C)

Figure S5. The performance of the ANN models on balanced datasets, in which negative samples, as many as positive samples, are randomly selected under different thresholds. (A) Six measurements; (B) The ROC curves; (C) The PR curves.

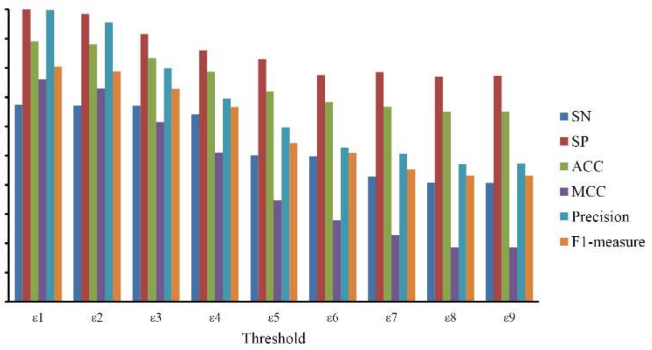

(A)

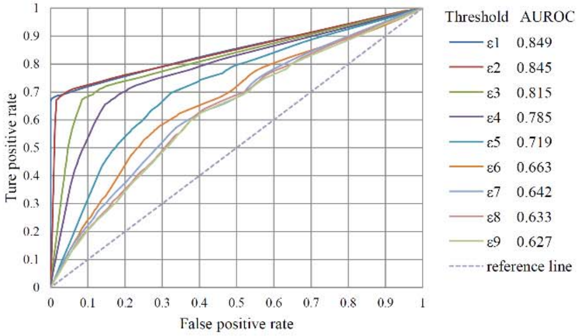

(B)

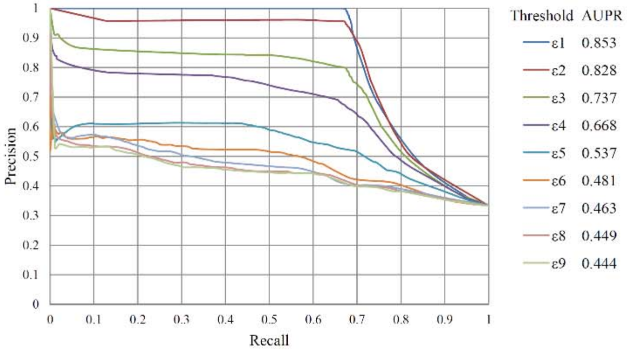

(C)

Figure S6. The performance of the ANN models on imbalanced datasets, in which negative samples, twice as many as positive samples, are randomly selected under different thresholds. (A) Six measurements; (B) The ROC curves; (C) The PR curves.

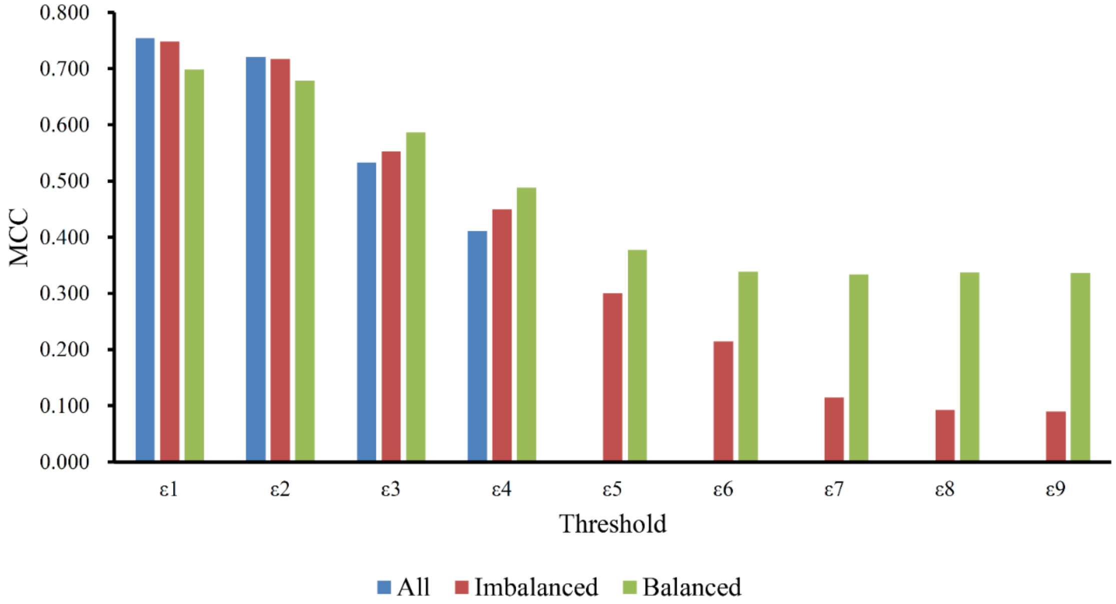

Figure S7. The MCCs yielded by the SVM models on three types of datasets. 'All' means that all negative samples under the given the threshold are selected, 'Imbalanced' indicates that negative samples, twice as many as positive samples, under the given threshold are randomly selected, 'Balanced' indicates that negative samples, as many as positive samples, under the given threshold are randomly selected.

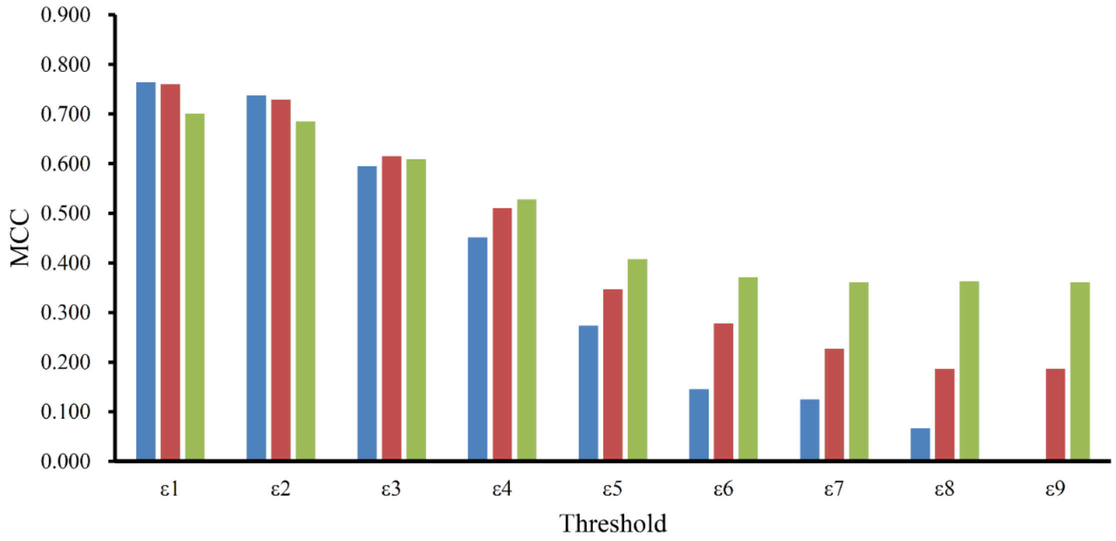

Figure S8. The MCCs yielded by the ANN models on three types of datasets. ‘All’ means that all negative samples under the given the threshold are selected, ‘Imbalanced’ indicates that negative samples, twice as many as positive samples, under the given threshold are randomly selected, ‘Balanced’ indicates that negative samples, as many as positive samples, under the given threshold are randomly selected.
